# A 4‐year‐old boy with a ventricular mass

**DOI:** 10.1111/bpa.13081

**Published:** 2022-06-14

**Authors:** Jun Zhou, Kexuan Qu, Mengxing Lv, Yan Gao, Lin Zhang, Ling Duan, Zhixiang A, Hongfang Wu, Yucheng Xie

**Affiliations:** ^1^ Department of Pathology Kunming Children's Hospital Kunming P.R. China; ^2^ Medical Imaging Department Kunming Children's Hospital Kunming P.R. China; ^3^ Clinical Lab Yunnan Province Second People's Hospital Kunming P.R. China

**Keywords:** corpus callosum, molecular neuro‐oncology, myxoid glioneuronal tumor, *PDGFRA*

## 1 CLINICAL HISTORY

**FIGURE 1:**
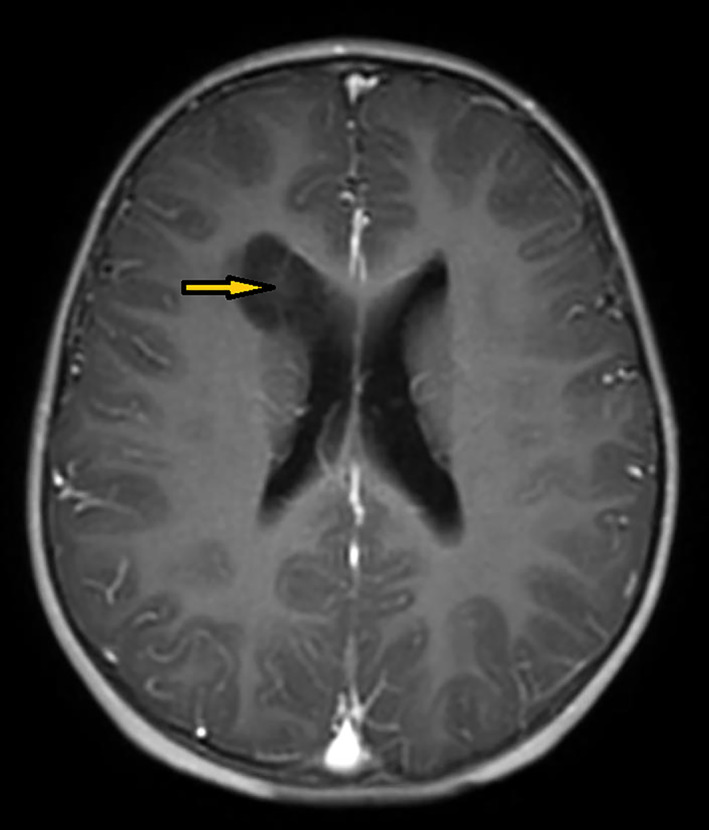
Axial T1‐weighted contrast enhanced magnetic resonance imaging shows the low intensity mass was located on the front edge of the right brain ventricle, with a few darker strands and no enhancement (arrowhead).

A 4‐year‐old boy was admitted to our hospital because of fever and convulsions, with a body temperature of 39.4°C. No specific abnormalities were found in immunochemistry, urine, or fecal analyzes, except for elevated serum neuron‐specific enolase (21.45 ng/ml, normal range: 0–16.3). Computed tomography showed what might be an enlarged frontal boundary of the right ventricle with calcified foci. On magnetic resonance imaging, this seemed to correspond to a T1 low intensity mass, measuring 2.5 × 2.0 × 2.1 cm, with a few darker strands and no enhancement (Figure 1). A preliminary diagnosis of a low grade glial/glioneuronal neoplasm was made. The tumor was completely removed, with no complications.

BOX 1 **Slide scan**

Access the whole slide scan at http://image.upmc.edu:8080/NeuroPathology/BPA/BPA‐22‐02‐047.svs/view.apml?

## 2 FINDINGS

**FIGURE 2:**
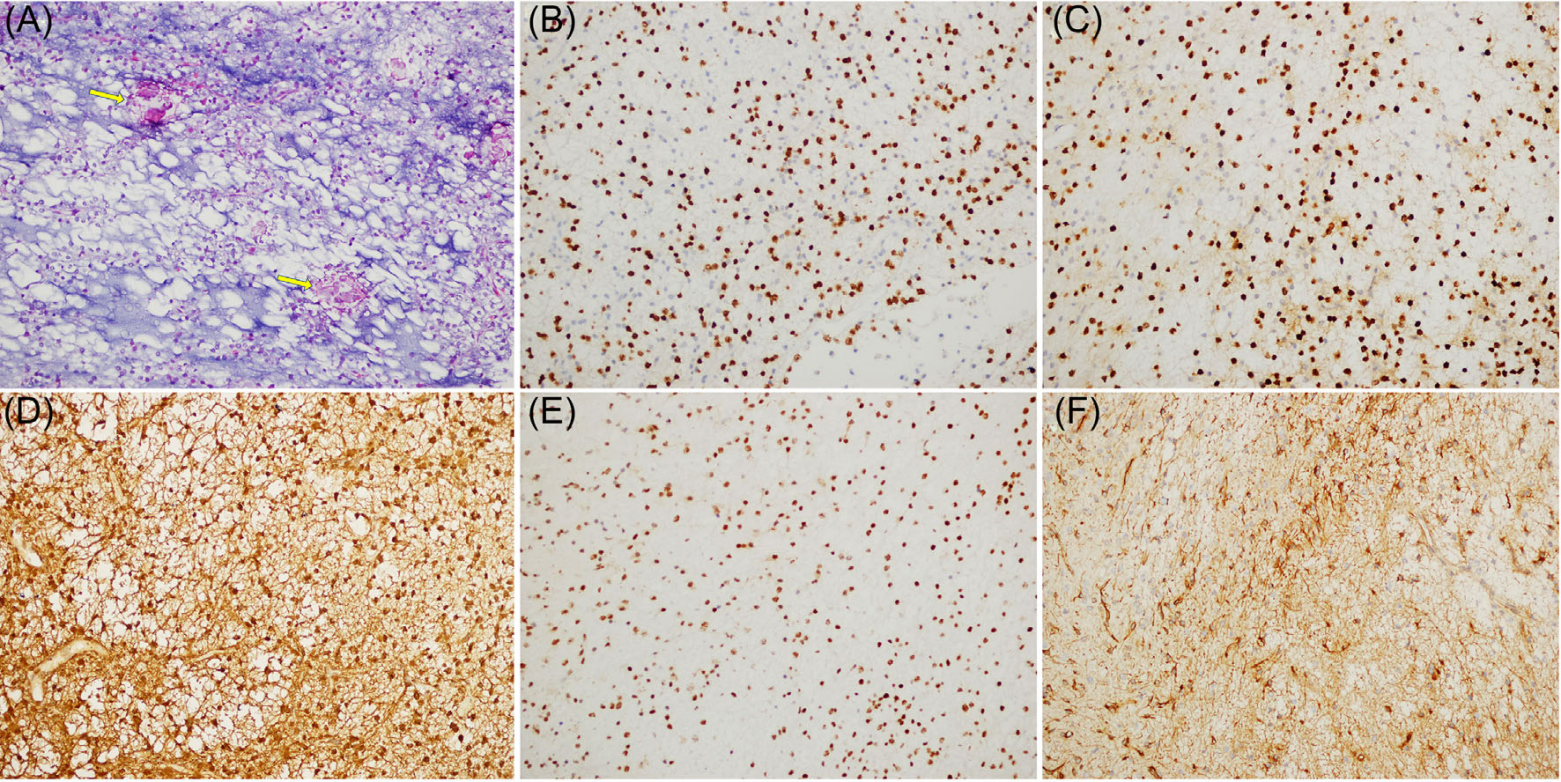
(A) The neoplasm composed of oligodendroglial‐like tumor cells with round to ovoid nuclei and scant cytoplasm immersed in a mucus rich extracellular matrix. Focal microcalcifications were present (arrowheads) (H&E, magnification ×200). (B) Immunoreactive for Olig2 (magnification ×200). (C) Immunoreactive for Sox10 (magnification ×200). (D) Immunoreactive for GFAP (magnification ×200). (E) Immunoreactive for ATRX (magnification ×200). (F) Immunoreactive for Nestin (magnification ×200)

Histopathological examination showed a low‐grade neoplasm composed of oligodendroglial‐like tumor cells with round to ovoid nuclei and scant cytoplasm immersed in a mucus‐rich extracellular matrix. Focal microcalcifications were also observed. No mitoses, microvascular proliferation or necrosis were observed (Figure 2A, Box 1). Immunohistochemical staining demonstrated Olig2 (Figure 2B), Sox10 (Figure 2C), GFAP (Figure 2D), ATRX (Figure 2E), and nestin (Figure 2F) expression.

## 3 DIAGNOSIS

Myxoid glioneuronal tumor (CNS WHO grade 1).

Targeted next‐generation sequencing analysis using a 599 gene panel revealed *PDGFRA* p.K385L (rate: 38.76%) and *NSD2* p.L581H (rate: 2.02%) mutations.

## 4 DISCUSSION

Myxoid glioneuronal tumor (MGNT) is a newly defined glioneuronal tumor in the 2021 WHO Classification of Tumors of the central nervous system, usually found in the septal area (nucleus accumbens or cavum septum pellucidum) and sometimes in the surrounding white matter or corpus callosum. MGNT can occur across a wide age range (6–65 years) [1], our patient, 4 years old, being the youngest patient reported at present. Clinical manifestations are variable, including headaches, vomiting, seizures, and behavioral disorders. In terms of its histological appearance, MGNT usually exhibits oligodendrocyte‐like tumor cells immersed in a prominent myxoid matrix. Floating neurons, neurocytic rosettes, and/or perivascular neuropil resembling findings typical of dysembrioplastic neuroepithelial tumor (DNET) or rosette‐forming glioneuronal tumor (RGNT) have also been observed. Mitotic activity is low or absent (<1/10 under high‐magnification view). Eosinophilic granular bodies, Rosenthal fibers, and microcalcifications typical of other low grade glioneuronal tumor are generally absent. It is considered a CNS WHO grade 1 tumor [2]. Examples of this tumors were previously reported under the term “dysembryoplastic neuroepithelial tumor of the septum” [3]. In 2018, Solomon et al. described mutations at codon p.K385 of *PDGFRA*, typically a dinucleotide mutation at codon 385 of the *PDGFRA* oncogene replacing lysine with either leucine or isoleucine (p.K385L/I), which appear to be highly characteristic for this tumor [3]. MGNT lacks *PIK3CA/PIK3R1* alterations or *BRAF/FGFR1* mutations which are characteristic of the majority of RGNT and DNET, respectively. Copy number profile is generally balanced. MGNT has a distinct DNA methylation profile, which together with the mutational pattern may be critical in some cases in distinguishing it from other central nervous system tumors.

In our case, the diagnosis of MGNT was suspected based on its histological appearance and confirmed by the molecular analysis. Targeted next‐generation sequencing analysis revealed *PDGFRA* p.K385L mutation. An additional gene mutation was observed in this case, namely, *NSD2* p.L581H (*NSD2*:NM_133330:exon10:c.1742T>A:p.L581H), also referred to as the MMSET or WHSC1. *NSD2* is located on chromosome pair 4 and encodes nuclear receptor‐binding SET domain protein 2. Specifically, *NSD2* catalyzes the methylation of the lysine site on the histone protein, thereby promoting the occurrence and progression of tumors through interactions with other proteins or regulation of target genes. However, as *NSD2* rarely been reported in CNS tumors, its significance in the occurrence and progression of MGNT is unclear.

## AUTHOR CONTRIBUTIONS

Jun Zhou and Kexuan Qu designed the study and drafted the manuscript; Yucheng Xie made the diagnosis; Yan Gao and Mengxing Lv reviewed the specimen; Ling Duan and Zhixiang A conducted the IHC and molecular analysis; Hong‐Fang Wu provided clinical data; Lin Zhang provided imaging data; all authors took part in writing the manuscript and approved the final, submitted version.

## FUNDING INFORMATION

This study was funded by Kunming Health Science and Technology Personnel Training Project and “Ten Hundred Thousand” Project (fund number: 2021‐SW (province)‐024).

## CONFLICT OF INTEREST

The authors declare that they have no conflicts of interest.

## ETHICAL STANDARDS AND PATIENT CONSENT

Ethical approval was obtained from the ethical committee from the Kunming Children's Hospital, Kunming, China.

## PATIENT CONSENT

Written informed consent was obtained from the patient's parents.

## Data Availability

The data on investigation results of this case are available from the corresponding author upon reasonable request.
