# Correction to “A 4‐year‐old boy with a ventricular mass”

**DOI:** 10.1111/bpa.13266

**Published:** 2024-04-22

Zhou J , Qu K , Lv M , Gao Y , Zhang L , Duan L , et al. A 4‐year‐old boy with a ventricular mass. Brain Pathology. 2022; 32(5):e13081. 10.1111/bpa.13081 35699689 PMC9425003

Figure 1 of this article has been published in another article shortly before by a different group of authors from the same institute elsewhere [1]. The authors have confirmed that they had access to the MR images and the histology results of their study from the hospitals public database and were not aware of the other group's publication. Although both articles describe the same patient, and partially overlapping results, yet the content is sufficiently different not to be considered redundant.

The authors have obtained retrospective permission for the reproduction of this image from the Chinese Journal of Contemporary Neurology & Neurosurgery. The updated caption of Figure 1 with the copyright statement is as follows:

**FIGURE 1** Axial T1‐weighted contrast enhanced magnetic resonance imaging shows the low intensity mass was located on the front edge of the right brain ventricle, with a few darker strands and no enhancement (arrowhead). Reprinted with permission, Copyright 2022, Chinese Journal of Contemporary Neurology & Neurosurgery [1].

The authors also wished to make a correction to the affiliation of the co‐authors, Kexuan Qu and Mengxing Lv.

The correct affiliation is: Department of Pathology, Kunming Children's Hospital, Kunming, P.R. China and Department of Blood Transfusion, Kunming Children's Hospital, Kunming, P. R. China
